# Undeca­carbonyl-1κ^3^ *C*,2κ^4^ *C*,3κ^4^ *C*-[tris­(2-chloro­eth­yl) phosphite-1κ*P*]-*triangulo*-triruthenium(0)

**DOI:** 10.1107/S1600536810033891

**Published:** 2010-09-04

**Authors:** Omar bin Shawkataly, Syed Sauban Ghani, Rajabathar Jothiramalingam, Chin Sing Yeap, Hoong-Kun Fun

**Affiliations:** a Chemical Sciences Programme, School of Distance Education, Universiti Sains Malaysia, 11800 USM, Penang, Malaysia; b X-ray Crystallography Unit, School of Physics, Universiti Sains Malaysia, 11800 USM, Penang, Malaysia

## Abstract

In the title *triangulo*-triruthenium compound, [Ru_3_(C_6_H_12_Cl_3_O_3_P)(CO)_11_], one equatorial carbonyl ligand is substituted by a monodentate phosphite ligand, leaving one equatorial and two axial carbonyl ligands on one Ru atom. The remaining two Ru atoms each carry two equatorial and two axial terminal carbonyl ligands. In the crystal structure, the mol­ecules are linked into a one-dimensional column along [100] by inter­molecular C—H⋯O hydrogen bonds.

## Related literature

For general background to *triangulo*-triruthenium derivatives, see: Bruce *et al.* (1985 ▶, 1988*a* ▶,*b* ▶). For the synthesis, see: Bruce *et al.* (1987 ▶). For related structures, see: Shawkataly *et al.* (1991 ▶, 2010 ▶). For the stability of the temperature controller used in the data collection, see: Cosier & Glazer (1986 ▶).
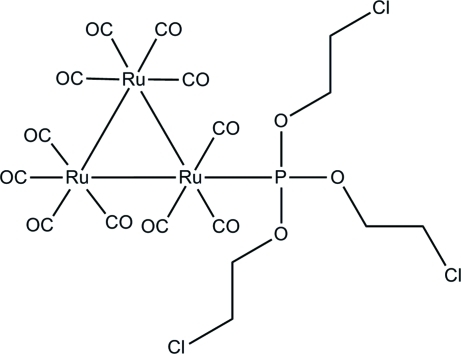


## Experimental

### 

#### Crystal data

[Ru_3_(C_6_H_12_Cl_3_O_3_P)(CO)_11_]
*M* *_r_* = 880.80
Triclinic, 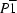

*a* = 7.8592 (9) Å
*b* = 12.5979 (14) Å
*c* = 14.8393 (17) Å
α = 109.442 (3)°
β = 93.791 (3)°
γ = 90.763 (3)°
*V* = 1381.4 (3) Å^3^
*Z* = 2
Mo *K*α radiation
μ = 2.03 mm^−1^
*T* = 100 K
0.20 × 0.19 × 0.03 mm

#### Data collection

Bruker APEXII DUO CCD diffractometer
Absorption correction: multi-scan (*SADABS*; Bruker, 2001 ▶) *T*_min_ = 0.691, *T*_max_ = 0.936
32458 measured reflections
11981 independent reflections
9935 reflections with *I* > 2σ(*I*)
*R*_int_ = 0.037

#### Refinement

*R*[*F* ^2^ > 2σ(*F* ^2^)] = 0.030
*wR*(*F* ^2^) = 0.120
*S* = 1.07
11981 reflections
343 parameters
H-atom parameters constrained
Δρ_max_ = 1.36 e Å^−3^
Δρ_min_ = −1.36 e Å^−3^

### 

Data collection: *APEX2* (Bruker, 2007 ▶); cell refinement: *SAINT* (Bruker, 2007 ▶); data reduction: *SAINT*; program(s) used to solve structure: *SHELXTL* (Sheldrick, 2008 ▶); program(s) used to refine structure: *SHELXTL*; molecular graphics: *SHELXTL*; software used to prepare material for publication: *SHELXTL* and *PLATON* (Spek, 2009 ▶).

## Supplementary Material

Crystal structure: contains datablocks global, I. DOI: 10.1107/S1600536810033891/hy2343sup1.cif

Structure factors: contains datablocks I. DOI: 10.1107/S1600536810033891/hy2343Isup2.hkl

Additional supplementary materials:  crystallographic information; 3D view; checkCIF report

## Figures and Tables

**Table 1:** Hydrogen-bond geometry (Å, °)

*D*—H⋯*A*	*D*—H	H⋯*A*	*D*⋯*A*	*D*—H⋯*A*
C17—H17*B*⋯O4^i^	0.97	2.58	3.297 (4)	131
C17—H17*B*⋯O5^ii^	0.97	2.54	3.307 (4)	136

Symmetry codes: (i) 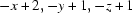; (ii) 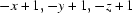.

**Fig. 1.**
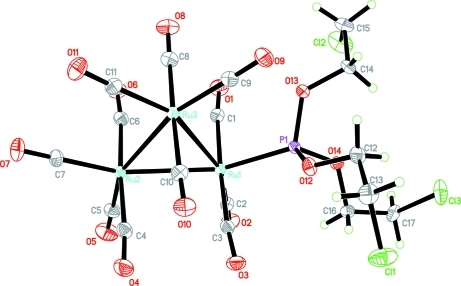
The molecular structure of the title compound with 50% probability ellipsoids for non-H atoms.

**Fig. 2.**
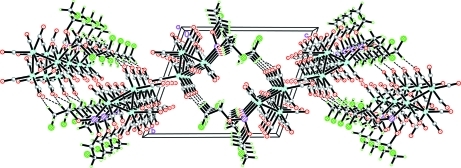
The crystal packing of the title compound, viewed down the a axis, showing the molecules linked into one-dimensional columns along the a axis.

## supplementary crystallographic information

### Comment

Syntheses and structures of substituted *triangulo*-triruthenium clusters have been of interest to researchers due to observed structural variations and their potential catalytic activity. A large number of substituted derivatives, Ru_3_(CO)_12-n_*L*_n_ (*L*= group 15 ligands), have been reported (Bruce *et al.*, 1985, 1988*a*,b). As part of our ongoing studies on phosphite substituted *triangulo-*triruthenium clusters (Shawkataly *et al.*, 1991, 2010), herein we report the structure of the title compound.

In the title compound (Fig. 1), a monodentate phosphite ligand has replaced a single carbonyl ligand of the Ru_3_ triangle. The monodentate phosphite ligand is bonded equatorially to the Ru1 atoms of the *triangulo*-triruthenium unit. Thus, the Ru2 and Ru3 atoms each carry two equatorial and two axial terminal carbonyl ligands, while the phosphite-bonded Ru1 atom binds one equatorial and two axial terminal carbonyl ligands.

In the crystal structure, the molecules are linked into a one-dimensional column along [1 0 0] by intermolecular C—H···O hydrogen bonds (Fig. 2, Table 1).

### Experimental

All the manipulations were performed under a dry oxygen-free nitrogen atmosphere using standard Schlenk techniques. THF was dried over sodium wire and freshly distilled from sodium benzophenone ketyl solution. The title compound was prepared by mixing Ru_3_(CO)_12_ (Aldrich) and P(OCH_2_CH_2_Cl)_3_ (Maybridge) in a 1:1 molar ratio in THF at 40°C. Diphenylketyl radical anion initiator of about 0.2 ml (synthesized as per the method of Bruce *et al.*, 1987) was introduced into the reaction mixture under a current of nitrogen. After stirring of 15 min, the solvent was removed under vacuum. Separation of the product in a pure form was done by column chromatography (Florisil, 100–200 mesh; eluant, dichloromethane: hexane). Crystals suitable for X-ray diffraction were grown by slow diffusion of CH_3_OH into the CH_2_Cl_2_ solution.

### Refinement

H atoms were positioned geometrically and refined using a riding model, with C—H = 0.97 Å and *U*_iso_(H) = 1.2*U*_eq_(C). The maximum and minimum residual electron density peaks of 1.36 and -1.36 eÅ^-3^ were located 1.32 and 0.81 Å from the C8 and Ru3 atoms, respectively.

### Crystal data

**Table:**

[Ru_3_(C_6_H_12_Cl_3_O_3_P)(CO)_11_]	*Z* = 2
*M**_r_* = 880.80	*F*(000) = 848
Triclinic, *P*1	*D*_x_ = 2.117 Mg m^−^^3^
Hall symbol: -P 1	Mo *K*α radiation, λ = 0.71073 Å
*a* = 7.8592 (9) Å	Cell parameters from 9961 reflections
*b* = 12.5979 (14) Å	θ = 2.6–34.9°
*c* = 14.8393 (17) Å	µ = 2.03 mm^−^^1^
α = 109.442 (3)°	*T* = 100 K
β = 93.791 (3)°	Plate, orange
γ = 90.763 (3)°	0.20 × 0.19 × 0.03 mm
*V* = 1381.4 (3) Å^3^	

### Data collection

**Table:**

Bruker APEXII DUO CCD diffractometer	11981 independent reflections
Radiation source: fine-focus sealed tube	9935 reflections with *I* > 2σ(*I*)
graphite	*R*_int_ = 0.037
φ and ω scans	θ_max_ = 35.0°, θ_min_ = 1.7°
Absorption correction: multi-scan (*SADABS*; Bruker, 2001)	*h* = −12→11
*T*_min_ = 0.691, *T*_max_ = 0.936	*k* = −20→20
32458 measured reflections	*l* = −23→23

### Refinement

**Table:**

Refinement on *F*^2^	Primary atom site location: structure-invariant direct methods
Least-squares matrix: full	Secondary atom site location: difference Fourier map
*R*[*F*^2^ > 2σ(*F*^2^)] = 0.030	Hydrogen site location: inferred from neighbouring sites
*wR*(*F*^2^) = 0.120	H-atom parameters constrained
*S* = 1.07	*w* = 1/[σ^2^(*F*_o_^2^) + (0.0712*P*)^2^] where *P* = (*F*_o_^2^ + 2*F*_c_^2^)/3
11981 reflections	(Δ/σ)_max_ = 0.001
343 parameters	Δρ_max_ = 1.36 e Å^−^^3^
0 restraints	Δρ_min_ = −1.36 e Å^−^^3^

### Special details

**Table:**

Experimental. The crystal was placed in the cold stream of an Oxford Cryosystems Cobra open-flow nitrogen cryostat (Cosier & Glazer, 1986) operating at 100.0 (1) K.

### Fractional atomic coordinates and isotropic or equivalent isotropic displacement parameters (Å^2^)

**Table:**

	*x*	*y*	*z*	*U*_iso_*/*U*_eq_	
Ru1	0.71803 (3)	0.328125 (16)	0.260791 (13)	0.01152 (5)	
Ru2	0.70797 (3)	0.443891 (16)	0.125659 (14)	0.01282 (5)	
Ru3	0.90548 (3)	0.247218 (16)	0.093890 (14)	0.01285 (5)	
Cl1	1.27297 (12)	0.29528 (8)	0.52857 (7)	0.0393 (2)	
Cl2	0.44453 (13)	−0.08078 (8)	0.26311 (8)	0.0408 (2)	
Cl3	0.87744 (12)	0.12562 (8)	0.59050 (6)	0.03316 (18)	
P1	0.81185 (9)	0.19414 (5)	0.32030 (5)	0.01243 (11)	
O1	0.4173 (3)	0.1665 (2)	0.15829 (18)	0.0261 (5)	
O2	0.4589 (3)	0.4615 (2)	0.39455 (16)	0.0272 (5)	
O3	1.0116 (3)	0.48098 (19)	0.38775 (16)	0.0243 (4)	
O4	1.0024 (4)	0.6040 (2)	0.24393 (19)	0.0312 (5)	
O5	0.4624 (4)	0.6254 (2)	0.2229 (2)	0.0353 (6)	
O6	0.4026 (3)	0.2931 (2)	0.01285 (18)	0.0264 (5)	
O7	0.8072 (3)	0.4917 (2)	−0.05275 (17)	0.0285 (5)	
O8	0.6085 (3)	0.0883 (2)	−0.02193 (18)	0.0286 (5)	
O9	1.0676 (3)	0.03923 (18)	0.11984 (17)	0.0237 (4)	
O10	1.2121 (3)	0.3946 (2)	0.21192 (18)	0.0246 (4)	
O11	1.0379 (3)	0.2483 (2)	−0.09539 (17)	0.0296 (5)	
O12	1.0146 (3)	0.20752 (17)	0.34233 (15)	0.0185 (4)	
O13	0.7651 (3)	0.06914 (16)	0.25139 (14)	0.0167 (3)	
O14	0.7425 (3)	0.18383 (16)	0.41609 (13)	0.0156 (3)	
C1	0.5297 (4)	0.2259 (2)	0.1906 (2)	0.0170 (5)	
C2	0.5606 (4)	0.4129 (2)	0.34655 (19)	0.0176 (5)	
C3	0.9063 (4)	0.4263 (2)	0.33632 (19)	0.0162 (4)	
C4	0.8986 (4)	0.5401 (2)	0.2013 (2)	0.0197 (5)	
C5	0.5525 (4)	0.5573 (2)	0.1872 (2)	0.0213 (5)	
C6	0.5187 (4)	0.3433 (2)	0.0568 (2)	0.0186 (5)	
C7	0.7704 (4)	0.4744 (2)	0.0129 (2)	0.0190 (5)	
C8	0.7114 (4)	0.1509 (2)	0.0235 (2)	0.0195 (5)	
C9	1.0079 (4)	0.1172 (2)	0.1112 (2)	0.0176 (5)	
C10	1.0934 (4)	0.3452 (2)	0.1710 (2)	0.0178 (5)	
C11	0.9900 (4)	0.2496 (2)	−0.0249 (2)	0.0191 (5)	
C12	1.1191 (4)	0.1307 (2)	0.3739 (2)	0.0201 (5)	
H12A	1.1351	0.0632	0.3201	0.024*	
H12B	1.0627	0.1090	0.4217	0.024*	
C13	1.2881 (4)	0.1874 (3)	0.4157 (2)	0.0251 (6)	
H13A	1.3346	0.2200	0.3715	0.030*	
H13B	1.3661	0.1316	0.4238	0.030*	
C14	0.7869 (4)	−0.0323 (2)	0.2750 (2)	0.0206 (5)	
H14A	0.7794	−0.0160	0.3433	0.025*	
H14B	0.8982	−0.0619	0.2585	0.025*	
C15	0.6486 (4)	−0.1176 (2)	0.2195 (2)	0.0241 (6)	
H15A	0.6760	−0.1907	0.2237	0.029*	
H15B	0.6449	−0.1235	0.1525	0.029*	
C16	0.7696 (4)	0.2759 (2)	0.50584 (19)	0.0183 (5)	
H16A	0.6951	0.3368	0.5060	0.022*	
H16B	0.8869	0.3046	0.5150	0.022*	
C17	0.7303 (4)	0.2308 (3)	0.5846 (2)	0.0211 (5)	
H17A	0.6149	0.1984	0.5727	0.025*	
H17B	0.7374	0.2919	0.6455	0.025*	

### Atomic displacement parameters (Å^2^)

**Table:**

	*U*^11^	*U*^22^	*U*^33^	*U*^12^	*U*^13^	*U*^23^
Ru1	0.01204 (9)	0.01129 (8)	0.01162 (8)	0.00113 (6)	0.00190 (6)	0.00414 (6)
Ru2	0.01309 (9)	0.01271 (8)	0.01366 (9)	0.00183 (6)	0.00155 (6)	0.00559 (6)
Ru3	0.01169 (9)	0.01307 (9)	0.01344 (9)	0.00218 (6)	0.00272 (6)	0.00355 (6)
Cl1	0.0261 (4)	0.0352 (4)	0.0420 (5)	0.0005 (3)	−0.0078 (4)	−0.0045 (4)
Cl2	0.0277 (4)	0.0341 (4)	0.0615 (6)	0.0012 (3)	0.0209 (4)	0.0140 (4)
Cl3	0.0348 (4)	0.0420 (4)	0.0343 (4)	0.0164 (4)	0.0094 (3)	0.0264 (4)
P1	0.0123 (3)	0.0118 (2)	0.0135 (3)	0.0009 (2)	0.0010 (2)	0.0046 (2)
O1	0.0173 (10)	0.0285 (11)	0.0295 (11)	−0.0045 (8)	−0.0009 (8)	0.0065 (9)
O2	0.0225 (11)	0.0326 (12)	0.0220 (10)	0.0072 (9)	0.0069 (8)	0.0018 (9)
O3	0.0253 (11)	0.0209 (9)	0.0222 (10)	−0.0057 (8)	−0.0020 (8)	0.0023 (8)
O4	0.0335 (14)	0.0245 (11)	0.0345 (13)	−0.0079 (10)	−0.0086 (11)	0.0108 (10)
O5	0.0309 (14)	0.0253 (11)	0.0446 (15)	0.0083 (10)	0.0135 (11)	0.0026 (10)
O6	0.0194 (11)	0.0268 (11)	0.0283 (11)	−0.0004 (9)	−0.0045 (9)	0.0044 (9)
O7	0.0285 (12)	0.0391 (13)	0.0258 (11)	0.0016 (10)	0.0059 (9)	0.0205 (10)
O8	0.0215 (11)	0.0243 (10)	0.0319 (12)	0.0010 (9)	0.0000 (9)	−0.0013 (9)
O9	0.0224 (11)	0.0202 (9)	0.0298 (11)	0.0062 (8)	0.0044 (8)	0.0093 (8)
O10	0.0159 (10)	0.0268 (11)	0.0281 (11)	−0.0014 (8)	0.0003 (8)	0.0054 (9)
O11	0.0287 (12)	0.0397 (13)	0.0222 (10)	0.0015 (10)	0.0085 (9)	0.0117 (9)
O12	0.0127 (8)	0.0203 (9)	0.0260 (10)	0.0015 (7)	0.0007 (7)	0.0125 (8)
O13	0.0218 (10)	0.0126 (7)	0.0158 (8)	−0.0002 (7)	0.0008 (7)	0.0050 (6)
O14	0.0175 (9)	0.0153 (8)	0.0138 (8)	−0.0015 (7)	0.0026 (7)	0.0046 (6)
C1	0.0152 (11)	0.0186 (11)	0.0176 (11)	0.0021 (9)	0.0017 (9)	0.0063 (9)
C2	0.0178 (12)	0.0191 (11)	0.0164 (10)	0.0013 (9)	0.0015 (9)	0.0064 (9)
C3	0.0179 (12)	0.0143 (10)	0.0170 (10)	−0.0007 (9)	0.0031 (9)	0.0056 (8)
C4	0.0179 (12)	0.0191 (11)	0.0235 (12)	0.0007 (9)	−0.0005 (10)	0.0093 (10)
C5	0.0212 (13)	0.0180 (11)	0.0234 (12)	0.0006 (10)	0.0048 (10)	0.0047 (10)
C6	0.0180 (12)	0.0169 (11)	0.0212 (12)	0.0027 (9)	0.0031 (9)	0.0065 (9)
C7	0.0171 (12)	0.0210 (11)	0.0215 (12)	0.0029 (9)	0.0018 (9)	0.0103 (10)
C8	0.0145 (11)	0.0198 (11)	0.0235 (12)	0.0027 (9)	0.0046 (9)	0.0056 (10)
C9	0.0148 (11)	0.0180 (11)	0.0188 (11)	0.0007 (9)	0.0043 (9)	0.0042 (9)
C10	0.0150 (11)	0.0198 (11)	0.0184 (11)	0.0023 (9)	0.0019 (9)	0.0059 (9)
C11	0.0174 (12)	0.0208 (11)	0.0183 (11)	0.0024 (9)	0.0034 (9)	0.0053 (9)
C12	0.0168 (12)	0.0188 (11)	0.0245 (12)	0.0054 (9)	−0.0009 (10)	0.0074 (10)
C13	0.0143 (12)	0.0297 (14)	0.0306 (15)	0.0042 (11)	0.0002 (11)	0.0092 (12)
C14	0.0266 (14)	0.0119 (10)	0.0230 (12)	0.0020 (9)	−0.0004 (11)	0.0057 (9)
C15	0.0252 (14)	0.0147 (11)	0.0292 (14)	0.0003 (10)	0.0065 (11)	0.0023 (10)
C16	0.0223 (13)	0.0160 (10)	0.0147 (10)	0.0022 (9)	0.0022 (9)	0.0022 (8)
C17	0.0204 (13)	0.0283 (13)	0.0156 (11)	0.0073 (11)	0.0020 (9)	0.0084 (10)

### Geometric parameters (Å, °)

**Table:**

Ru1—C2	1.905 (3)	O5—C5	1.131 (4)
Ru1—C3	1.945 (3)	O6—C6	1.135 (4)
Ru1—C1	1.946 (3)	O7—C7	1.120 (4)
Ru1—P1	2.2609 (7)	O8—C8	1.135 (4)
Ru1—Ru2	2.8431 (4)	O9—C9	1.136 (4)
Ru1—Ru3	2.8610 (4)	O10—C10	1.133 (4)
Ru2—C5	1.922 (3)	O11—C11	1.130 (4)
Ru2—C7	1.929 (3)	O12—C12	1.450 (3)
Ru2—C6	1.936 (3)	O13—C14	1.443 (3)
Ru2—C4	1.949 (3)	O14—C16	1.446 (3)
Ru2—Ru3	2.8622 (4)	C12—C13	1.493 (4)
Ru3—C9	1.919 (3)	C12—H12A	0.9700
Ru3—C11	1.934 (3)	C12—H12B	0.9700
Ru3—C8	1.945 (3)	C13—H13A	0.9700
Ru3—C10	1.950 (3)	C13—H13B	0.9700
Cl1—C13	1.784 (3)	C14—C15	1.509 (4)
Cl2—C15	1.779 (3)	C14—H14A	0.9700
Cl3—C17	1.790 (3)	C14—H14B	0.9700
P1—O13	1.589 (2)	C15—H15A	0.9700
P1—O14	1.600 (2)	C15—H15B	0.9700
P1—O12	1.601 (2)	C16—C17	1.507 (4)
O1—C1	1.122 (4)	C16—H16A	0.9700
O2—C2	1.148 (4)	C16—H16B	0.9700
O3—C3	1.137 (3)	C17—H17A	0.9700
O4—C4	1.135 (4)	C17—H17B	0.9700
			
C2—Ru1—C3	90.61 (12)	C12—O12—P1	124.95 (18)
C2—Ru1—C1	88.67 (12)	C14—O13—P1	126.29 (18)
C3—Ru1—C1	176.82 (11)	C16—O14—P1	120.43 (17)
C2—Ru1—P1	106.42 (9)	O1—C1—Ru1	173.4 (3)
C3—Ru1—P1	88.17 (8)	O2—C2—Ru1	176.4 (3)
C1—Ru1—P1	89.06 (8)	O3—C3—Ru1	173.4 (2)
C2—Ru1—Ru2	99.93 (8)	O4—C4—Ru2	173.8 (3)
C3—Ru1—Ru2	91.10 (8)	O5—C5—Ru2	178.8 (3)
C1—Ru1—Ru2	92.08 (8)	O6—C6—Ru2	172.8 (3)
P1—Ru1—Ru2	153.641 (19)	O7—C7—Ru2	179.6 (3)
C2—Ru1—Ru3	159.81 (8)	O8—C8—Ru3	173.9 (3)
C3—Ru1—Ru3	93.38 (8)	O9—C9—Ru3	178.8 (3)
C1—Ru1—Ru3	88.35 (8)	O10—C10—Ru3	173.8 (3)
P1—Ru1—Ru3	93.50 (2)	O11—C11—Ru3	178.3 (3)
Ru2—Ru1—Ru3	60.236 (9)	O12—C12—C13	109.3 (2)
C5—Ru2—C7	106.72 (13)	O12—C12—H12A	109.8
C5—Ru2—C6	90.60 (12)	C13—C12—H12A	109.8
C7—Ru2—C6	93.05 (12)	O12—C12—H12B	109.8
C5—Ru2—C4	89.78 (13)	C13—C12—H12B	109.8
C7—Ru2—C4	90.49 (12)	H12A—C12—H12B	108.3
C6—Ru2—C4	176.17 (12)	C12—C13—Cl1	112.2 (2)
C5—Ru2—Ru1	97.66 (9)	C12—C13—H13A	109.2
C7—Ru2—Ru1	155.59 (9)	Cl1—C13—H13A	109.2
C6—Ru2—Ru1	87.85 (9)	C12—C13—H13B	109.2
C4—Ru2—Ru1	88.32 (9)	Cl1—C13—H13B	109.2
C5—Ru2—Ru3	157.76 (9)	H13A—C13—H13B	107.9
C7—Ru2—Ru3	95.47 (9)	O13—C14—C15	108.5 (2)
C6—Ru2—Ru3	86.84 (8)	O13—C14—H14A	110.0
C4—Ru2—Ru3	91.37 (9)	C15—C14—H14A	110.0
Ru1—Ru2—Ru3	60.191 (8)	O13—C14—H14B	110.0
C9—Ru3—C11	103.01 (12)	C15—C14—H14B	110.0
C9—Ru3—C8	88.67 (12)	H14A—C14—H14B	108.4
C11—Ru3—C8	90.45 (12)	C14—C15—Cl2	112.2 (2)
C9—Ru3—C10	91.35 (12)	C14—C15—H15A	109.2
C11—Ru3—C10	92.68 (12)	Cl2—C15—H15A	109.2
C8—Ru3—C10	176.78 (12)	C14—C15—H15B	109.2
C9—Ru3—Ru1	101.60 (8)	Cl2—C15—H15B	109.2
C11—Ru3—Ru1	155.38 (9)	H15A—C15—H15B	107.9
C8—Ru3—Ru1	90.80 (9)	O14—C16—C17	107.3 (2)
C10—Ru3—Ru1	86.04 (8)	O14—C16—H16A	110.2
C9—Ru3—Ru2	161.17 (8)	C17—C16—H16A	110.2
C11—Ru3—Ru2	95.82 (9)	O14—C16—H16B	110.2
C8—Ru3—Ru2	91.17 (9)	C17—C16—H16B	110.2
C10—Ru3—Ru2	87.77 (9)	H16A—C16—H16B	108.5
Ru1—Ru3—Ru2	59.573 (8)	C16—C17—Cl3	110.5 (2)
O13—P1—O14	98.19 (10)	C16—C17—H17A	109.5
O13—P1—O12	108.50 (12)	Cl3—C17—H17A	109.5
O14—P1—O12	104.15 (11)	C16—C17—H17B	109.5
O13—P1—Ru1	113.83 (8)	Cl3—C17—H17B	109.5
O14—P1—Ru1	120.97 (8)	H17A—C17—H17B	108.1
O12—P1—Ru1	110.01 (8)		
			
C2—Ru1—Ru2—C5	1.98 (12)	C4—Ru2—Ru3—C9	88.2 (3)
C3—Ru1—Ru2—C5	−88.83 (12)	Ru1—Ru2—Ru3—C9	0.9 (3)
C1—Ru1—Ru2—C5	91.00 (12)	C5—Ru2—Ru3—C11	175.3 (3)
P1—Ru1—Ru2—C5	−176.92 (10)	C7—Ru2—Ru3—C11	−1.28 (12)
Ru3—Ru1—Ru2—C5	177.91 (9)	C6—Ru2—Ru3—C11	91.49 (12)
C2—Ru1—Ru2—C7	179.0 (2)	C4—Ru2—Ru3—C11	−91.90 (12)
C3—Ru1—Ru2—C7	88.2 (2)	Ru1—Ru2—Ru3—C11	−179.18 (9)
C1—Ru1—Ru2—C7	−92.0 (2)	C5—Ru2—Ru3—C8	84.8 (3)
P1—Ru1—Ru2—C7	0.1 (2)	C7—Ru2—Ru3—C8	−91.85 (13)
Ru3—Ru1—Ru2—C7	−5.1 (2)	C6—Ru2—Ru3—C8	0.91 (12)
C2—Ru1—Ru2—C6	−88.33 (12)	C4—Ru2—Ru3—C8	177.52 (12)
C3—Ru1—Ru2—C6	−179.15 (11)	Ru1—Ru2—Ru3—C8	90.24 (9)
C1—Ru1—Ru2—C6	0.68 (11)	C5—Ru2—Ru3—C10	−92.2 (3)
P1—Ru1—Ru2—C6	92.76 (9)	C7—Ru2—Ru3—C10	91.19 (12)
Ru3—Ru1—Ru2—C6	87.59 (9)	C6—Ru2—Ru3—C10	−176.05 (11)
C2—Ru1—Ru2—C4	91.53 (12)	C4—Ru2—Ru3—C10	0.56 (12)
C3—Ru1—Ru2—C4	0.72 (11)	Ru1—Ru2—Ru3—C10	−86.72 (8)
C1—Ru1—Ru2—C4	−179.46 (12)	C5—Ru2—Ru3—Ru1	−5.5 (2)
P1—Ru1—Ru2—C4	−87.38 (10)	C7—Ru2—Ru3—Ru1	177.90 (9)
Ru3—Ru1—Ru2—C4	−92.55 (9)	C6—Ru2—Ru3—Ru1	−89.33 (9)
C2—Ru1—Ru2—Ru3	−175.93 (9)	C4—Ru2—Ru3—Ru1	87.28 (9)
C3—Ru1—Ru2—Ru3	93.26 (8)	C2—Ru1—P1—O13	117.48 (13)
C1—Ru1—Ru2—Ru3	−86.91 (8)	C3—Ru1—P1—O13	−152.42 (12)
P1—Ru1—Ru2—Ru3	5.17 (4)	C1—Ru1—P1—O13	29.15 (12)
C2—Ru1—Ru3—C9	−168.0 (3)	Ru2—Ru1—P1—O13	−63.64 (10)
C3—Ru1—Ru3—C9	90.97 (12)	Ru3—Ru1—P1—O13	−59.14 (9)
C1—Ru1—Ru3—C9	−86.36 (12)	C2—Ru1—P1—O14	0.96 (13)
P1—Ru1—Ru3—C9	2.60 (9)	C3—Ru1—P1—O14	91.05 (12)
Ru2—Ru1—Ru3—C9	−179.70 (9)	C1—Ru1—P1—O14	−87.38 (12)
C2—Ru1—Ru3—C11	13.7 (3)	Ru2—Ru1—P1—O14	179.83 (8)
C3—Ru1—Ru3—C11	−87.4 (2)	Ru3—Ru1—P1—O14	−175.67 (9)
C1—Ru1—Ru3—C11	95.3 (2)	C2—Ru1—P1—O12	−120.51 (12)
P1—Ru1—Ru3—C11	−175.7 (2)	C3—Ru1—P1—O12	−30.41 (12)
Ru2—Ru1—Ru3—C11	2.0 (2)	C1—Ru1—P1—O12	151.16 (12)
C2—Ru1—Ru3—C8	−79.2 (3)	Ru2—Ru1—P1—O12	58.37 (10)
C3—Ru1—Ru3—C8	179.78 (11)	Ru3—Ru1—P1—O12	62.86 (9)
C1—Ru1—Ru3—C8	2.45 (12)	O13—P1—O12—C12	−49.8 (2)
P1—Ru1—Ru3—C8	91.41 (9)	O14—P1—O12—C12	54.1 (2)
Ru2—Ru1—Ru3—C8	−90.89 (9)	Ru1—P1—O12—C12	−174.9 (2)
C2—Ru1—Ru3—C10	101.4 (3)	O14—P1—O13—C14	−41.8 (3)
C3—Ru1—Ru3—C10	0.41 (11)	O12—P1—O13—C14	66.2 (3)
C1—Ru1—Ru3—C10	−176.92 (11)	Ru1—P1—O13—C14	−171.0 (2)
P1—Ru1—Ru3—C10	−87.96 (9)	O13—P1—O14—C16	174.3 (2)
Ru2—Ru1—Ru3—C10	89.74 (9)	O12—P1—O14—C16	62.8 (2)
C2—Ru1—Ru3—Ru2	11.7 (2)	Ru1—P1—O14—C16	−61.5 (2)
C3—Ru1—Ru3—Ru2	−89.33 (8)	P1—O12—C12—C13	−162.5 (2)
C1—Ru1—Ru3—Ru2	93.34 (8)	O12—C12—C13—Cl1	71.2 (3)
P1—Ru1—Ru3—Ru2	−177.703 (19)	P1—O13—C14—C15	148.7 (2)
C5—Ru2—Ru3—C9	−4.6 (4)	O13—C14—C15—Cl2	−71.2 (3)
C7—Ru2—Ru3—C9	178.8 (3)	P1—O14—C16—C17	−166.17 (19)
C6—Ru2—Ru3—C9	−88.4 (3)	O14—C16—C17—Cl3	64.2 (3)

### Hydrogen-bond geometry (Å, °)

**Table:**

*D*—H···*A*	*D*—H	H···*A*	*D*···*A*	*D*—H···*A*
C17—H17B···O4^i^	0.97	2.58	3.297 (4)	131
C17—H17B···O5^ii^	0.97	2.54	3.307 (4)	136

Symmetry codes: (i) −*x*+2, −*y*+1, −*z*+1; (ii) −*x*+1, −*y*+1, −*z*+1.
